# Surgical treatment of pancreaticojejunal stenosis after pancreaticoduodenectomy: case report

**DOI:** 10.1093/jscr/rjae239

**Published:** 2024-06-11

**Authors:** David Narvaez Salas, Estefania Roldan-Vasquez, Ricardo Negrete Ocampo, Romina Ballagan Escobar, Juan Roldan Crespo

**Affiliations:** Departamento de Cirugía General, Hospital Vozandes Quito, Quito 170521, Ecuador; Departamento de Cirugía General, Hospital Vozandes Quito, Quito 170521, Ecuador; Department of General Surgery, Beth Israel Deaconess Medical Center, Harvard Medical School, Boston, MA 02215, United States; School of Medicine, Colegio de Ciencias de la Salud, Universidad San Francisco de Quito, Quito 170901, Ecuador; Department of General Surgery, Beth Israel Deaconess Medical Center, Harvard Medical School, Boston, MA 02215, United States

**Keywords:** pancreatoduodenectomy, pancreato-jejunostomy stenosis, pancreatitis

## Abstract

Pancreaticoduodenectomy is established as the procedure of choice for malignant tumor pathologies of the head of the pancreas or ampulla, where the patient’s life prognosis is low. Complications after pancreaticoduodenectomy (e.g. pancreatic fistulas, hemorrhages, or intra-abdominal collection) are well described in the literature and are generally acute. However, there is still a small risk for late complications (e.g. pancreatitis, pancreatic insufficiency), and due to its low incidence, there has not been a consensus on the treatment. We present the case of an 18-year-old female with recurrent bouts of acute pancreatitis as a late complication of a pancreaticoduodenectomy plus pancreatojejunal anastomosis due to a pseudopapillary tumor of the pancreas. The complication was managed though surgical revision consisting of dilation and stent placement in the stenosis. The patient had an adequate postoperative evolution without further complications. Despite the advances in the surgical field, pancreaticoduodenectomy represents a highly complex surgery with high morbidity and mortality rates. The late complications of this surgery are under continuous study due to its low incidence associated with low patient survival.

## Introduction

Pancreatoduodenectomy (PD) remains the standard procedure for tumors of the ampulla and head of the pancreas. The resection involves the head and uncinate process of the pancreas, as well as the duodenum, first 15 cm of the jejunum, common bile duct, gallbladder, and proximal stomach. Pancreato-enteric reconstruction techniques continue to be the turning point in the prognosis of patients requiring PD [1].

Early complications of PD are well studied with a prevalence in the literature of up to 35% in relation to pancreatic fistulas and 3% in hemorrhages [2]. Thus, in patients with benign pancreatic pathologies, who require surgery and also have a good life prognosis, late complications can manifest and one of them is stenosis of the PJ anastomosis [2].

PJ stenosis is presented by recurrent and obstructive pancreatitis events [3]. Approximately less than 5% of patients undergoing PJ will present a late complication, and the median time from diagnosis from PD to diagnosis of PJ stenosis is 46 months [4].

Survival rates after PD have increased in recent years. Thus, late complications after this surgery are more frequent, but still not so well studied. Consequently, the natural history of PJ stenosis is not well documented; therefore, there are controversies regarding its treatment [3].

In the following case, we present the diagnosis and management of late PJ stenosis when presenting through recurrent pancreatitis.

## Clinical case

An 18-year-old female patient comes with severe abdominal pain. The patient has a history of pseudopapillary tumor of the pancreas managed by pancreatoduodenectomy with end-to-side pancreatojejunal anastomosis performed 24 months ago. In this instance, the patient went to the emergency room due to repetitive pain in the epigastrium, cramping, of moderate intensity, radiating to the lower back, accompanied by nausea and vomiting. She denies the presence of jaundice, acholia, or choluria.

**Figure 1:**
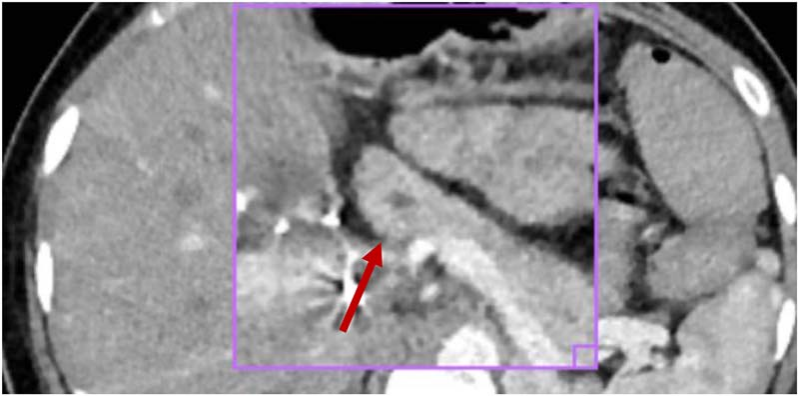
No inflammatory involvement of the body or pancreatic tail is observed; there is no alteration of the peripancreatic fat or collections at this level; visible wirsung canal measures 2.2 mm (red arrow).

The patient has a history of four episodes of acute pancreatitis which also presented with abdominal pain, accompanied by elevated pancreatic enzymes (Table 1). During his first event, imaging studies were performed where the tomography showed a normal main pancreatic duct (Fig. 1) with no evidence of collections.

**Table 1:** Markers of cholestasis and pancreatic enzymes in abdominal pain events.

**Acute pancreatitis events**	**Amylase (U/L)**	**Lipase (U/L)**	**GGT (U/L)**	**Total bilirubin (mg/dL)**	**Alkaline phosphatase (U/L)**
November 2019	58	1715	10	0,63	90	
February 2020	1442	3475	7	0,53	79	
December 2020	559	2539	294	—	166	
April 2021	954	7317	—	—	—	
August 2021	500	3708	—	—	—	

**Figure 2:**
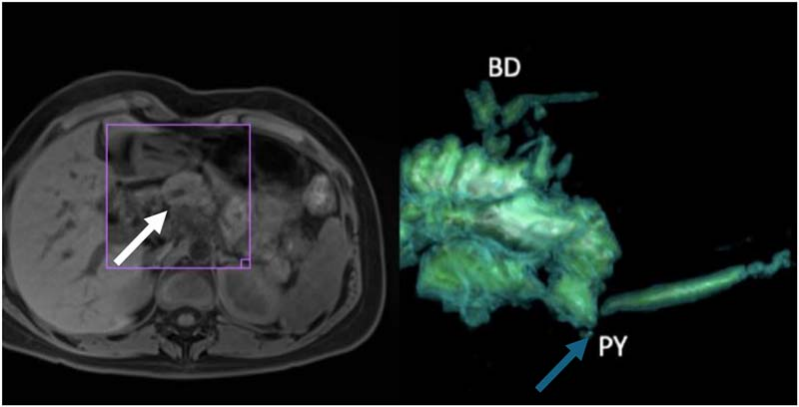
(A) Thickening of the tail of the pancreas with hyperintensity in diffusion sequence (white arrow), anteroposterior diameter 30 mm, inflammatory changes in the body and neck of the pancreas, discreet postcontrast enhancement, and findings suggestive of recurrent interstitial edematous acute pancreatitis; (B) 3D reconstruction with evidence of narrowing of the pancreatojejunal anastomosis (blue arrow).

After maintaining repetitive episodes of acute pancreatitis for 3 months after the first episode, it was decided to perform a magnetic resonance cholangiography, where a 6.1-mm pancreatic duct was determined (Fig. 2), suggestive of PJ stenosis.

Given the clinical and imaging results, it was decided to perform an endoscopic retrograde cholangiopancreatography (ERCP) which failed, and later, a second attempt was made by enteroscopy, where complete stenosis of the PJ anastomosis was visualized, for which it was decided not to proceed with cannulation.

**Figure 3:**
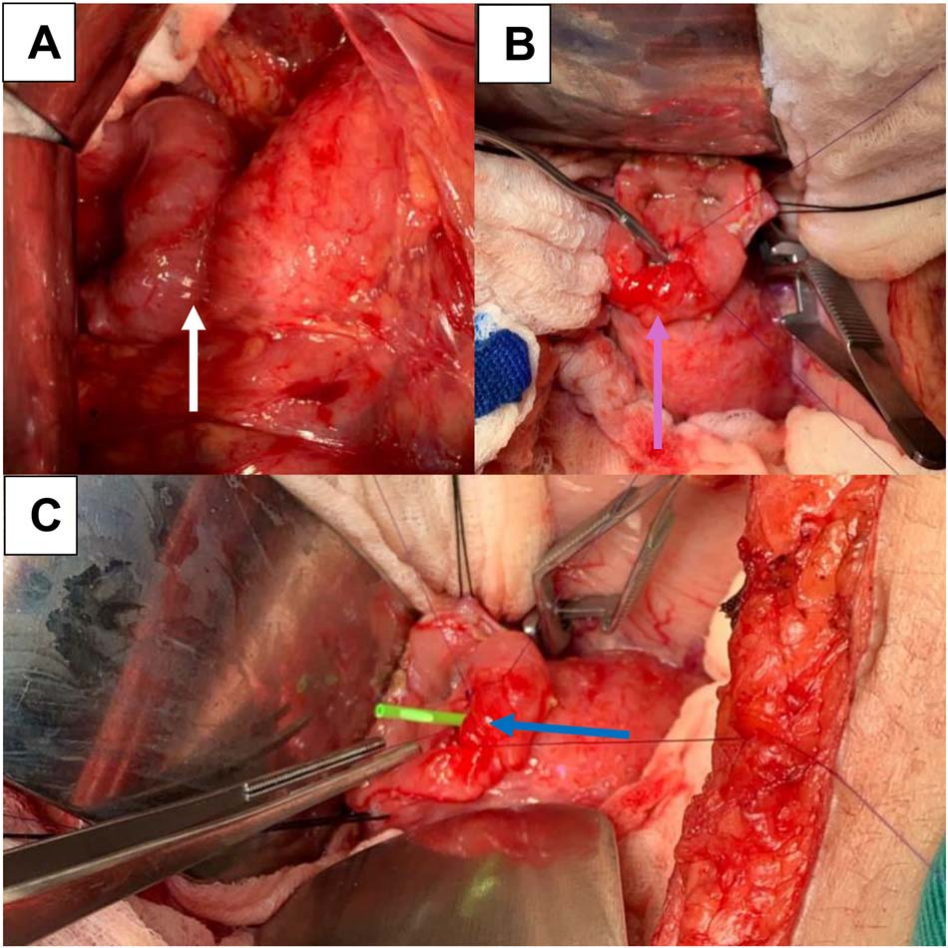
(A) Pancreato-jejunal anastomosis (white arrow); (B) enterotomy, mechanical dilation of the PJ anastomosis, and plasty (light blue arrow); (C) placement of plastic stent in PY anastomosis (blue arrow).

In the context of the patient, with two failed attempts at endoscopic dilation and due to persistence of symptoms, surgical resolution was decided. To correct the stenosis, a 2-mm-diameter jejunal enterotomy was performed at the level of the PJ anastomosis (Fig. 3A, B). Subsequently, progressive dilation was performed with metal dilators reaching a lumen diameter of 5 mm (Fig. 3B). Finally, a stent was placed in the anastomosis and a plasty was performed (Fig. 3C). The patency of the stenosis is confirmed by observing the passage of pancreatic fluid into the jejunum.

The patient presented a favorable postsurgical evolution without complications. Enteral feeding was resumed 24 h later, and hospital discharge was given 5 days after the procedure. Following her hospitalization, the patient has not presented other symptoms of acute pancreatitis.

## Discussion

Mortality related to PD has decreased, especially in benign etiologies; however, morbidity remains high, maintaining itself at ~40% [5]. PD complications are divided in early and late. Based on the literature, late complications can occur up to the first 5 years after the intervention. These can present through biliary stricture, cholangitis, pancreatitis, peptic ulcer, intestinal obstruction, and incisional hernia [5].

Brown *et al.* studied the factors associated with late complications such as a BMI > 30 kg/m^2^, surgical site infection, hospital readmission, and pylorus-sparing surgery. Some of these risk factors for the development of post-PD complications can be observed in our patient, such as pylorus-sparing surgery and hospital readmission.

The pathophysiology of PJ stenosis is not well defined. In relation to PJ stenosis, the patient may remain asymptomatic, develop symptoms associated with exocrine pancreatic insufficiency, or develop permanent severe abdominal pain [6]. According to the study conducted by Demirjian *et al.* [7], the PJ stenosis developed symptoms in 2% of the patients in an average time of 41 months and was associated with the previous presence of pancreatic fistulas, local inflammation, and subsequent fibrosis of the anastomosis. Reports of risk factors for the development of a PJ stenosis are inconsistent [8], but it is clear that the surgical technique of the anastomosis is not related to an increased risk [4].

The “gold” standard diagnosis for PJ stenosis is magnetic resonance cholangiography, where a reduction in the diameter of the main pancreatic duct at the level of the anastomosis must be found, accompanied by distal dilation [2]. Thus, the explanation for the development of recurrent AP is described by a mechanical obstructive process.

There are controversies regarding the therapeutic approach to PJ stenosis due to the low incidence. In the systematic review conducted by Vanbrugghe *et al.*, detail that treatment should be sequential, as was done in our patient. The approach must go from less to more invasive, starting with the endoscopic route, then endoscopic retrograde cholangiopancreatography or percutaneous treatments, and finally surgical intervention [2]. On several occasions, endoscopic intervention can be complicated by up to 25% [2], due to the altered posterior anatomy after PD, which increases the risk of perforation [9].

Surgical revision of a PJ stenosis is technically demanding. The Puestow surgical technique is the most frequently used; this consists of a longitudinal incision of the pancreatic duct, with subsequent anastomosis to the jejunal loop, with a reported hospital stay of 8–12 days [8]. Another technique to consider within the armamentarium is the performance of a new PJ anastomosis, where Seelig *et al.* [10] demonstrated morbidity rates of 33% and an average hospital stay of 15 days. If pancreatic inflammatory processes cause multiple adhesions and seal the PJ anastomosis, a transjejunalplasty is the procedure of choice [7].

Oida *et al.* [11] described in a case report a hybrid technique with surgical dilation of the pancreatic duct with subsequent placement of a stent in this anastomosis, similar to the technique we used in our patient, obtaining adequate results in terms of symptoms and recurrence of acute pancreatitis, with lower rates of morbidity and hospital stay.

## Conclusion

Since late complications after PD plus PJ anastomosis are rare, no consensus has yet been reached regarding their treatment. In our case, we demonstrated surgical management through dilation and stent placement, which obtained a positive evolution for the management of recurrent acute pancreatitis due to PJ stenosis.
